# Outreach Training and Supportive Supervision: A Package of Strategies That Improves the Quality of Malaria Services and Provides a Model for Monitoring and Evaluating Their Effective Implementation

**DOI:** 10.4269/ajtmh.23-0834

**Published:** 2024-02-06

**Authors:** MaryKelly S. McCarthy, S. Patrick Kachur

**Affiliations:** Heilbrunn Department of Population and Family Health, Columbia University Mailman School of Public Health, New York, New York

In this supplement, authors from the U.S. President’s Malaria Initiative (PMI) Impact Malaria Project present their experience with an approach to improving malaria case management and related services in 11 countries. Outreach Training and Supportive Supervision (OTSS) was initially developed to guide improvements in malaria diagnosis and has been adapted as a package of implementation strategies to support malaria case management and prevention interventions over multiple years. In this supplement, Impact Malaria partners demonstrate through an independent evaluation how the approach is adaptable to multiple settings and can enhance service quality in individual countries. They also apply supervisor checklists to create indicators of facility readiness and provider competency that can be a model for transforming information systems to track the impact of the malaria service investments made by countries and their partners more effectively. The results are relevant to malaria control efforts in high-burden contexts, elimination settings, and even countries certified as malaria free.

“Prompt and effective case management is the cornerstone of malaria programs.” Many of us have written that statement dozens of times and spoken it out from the podium at conferences. It remains as true today as it did following a Ministerial Conference in 1992.^1^ It was enshrined in the Abuja Declaration,^2^ and it persists in the current Global Technical Strategy.^3^ It holds for high-burden countries as well as those approaching elimination. Even countries that have been certified malaria free must maintain the ability to detect and treat imported cases with minimal delay, and guard against reintroduction of transmission. This clear and sustained commitment to ensuring that each malaria infection or illness is detected, diagnosed, and completely treated rests on a complex interplay of factors. Individual behaviors, cultural perceptions and practices, health system infrastructure and capacity, human resources, supply chains, health information systems, and financing don’t always converge in ways that make the accomplishment of quality case management a simple or seamless endeavor. Ensuring they do is a daunting challenge and still leaves too many cases undetected, underreported, and incompletely treated.^4^ In this issue of the *Journal*, contributors from the U.S. PMI Impact Malaria Project present work on the OTSS approach developed to support frontline health workers and district managers in their efforts to ensure universal access to quality malaria services.

There is considerable evidence of the benefits of prompt and effective malaria case management. It can reduce the duration of illness and disability, forestall progression to severe or fatal disease, shorten the duration of infection and risk of ongoing transmission, mitigate against the selection of drug-resistant parasites, and provide reliable data to track the impact of control and elimination efforts. Over recent decades, several innovations have transformed malaria case management. These include the adoption of artemisinin-based combination treatments (ACTs) as first-line drugs, the introduction and expansion of point-of-care diagnostic testing, and the opportunity to collect and act on meaningful trends in parasitologically confirmed cases through enhanced surveillance and response systems. These developments have improved *what* health workers and systems can provide. The OTSS represents a different sort of innovation, one that enhances *how* case management services are provided, monitored, and evaluated. In the language of implementation science, case management is a complex but technologically sound evidence-based intervention, and OTSS can be viewed as a package of implementation strategies that aim to optimize its delivery.

In the first paper of this series, Barat et al.^5^ characterize OTSS as a quality improvement approach that combines training, supervision, coaching, troubleshooting, action planning, and follow-up, and compile evidence and experience to support how the approach can improve the quality of malaria case management alongside systems supports, supplies, data collection, and use. Rather than stand-alone training workshops or onsite coaching or competency-based supervision, a strength of OTSS is the comprehensive and integrated support it provides health workers and managers. In an earlier systematic review,^6^ this sort of multifaceted strategy frequently showed better impact on quality of care than the component elements independent of one another. Another key feature of OTSS is its adaptability. It can and has been customized to meet the specific needs of different countries and health systems in the face of evolving challenges. Barat et al.^5^ describe the evolution of the OTSS strategy and its expansion from an original focus on diagnosis of uncomplicated malaria to a more comprehensive one that includes treatment, data collection, and reporting. They further describe examples of expanded approaches—termed OTSS Plus (OTSS+)—that also address related outcomes and interventions such as client satisfaction, data quality, management of severe malaria, delivery of chemoprevention, routine distribution of insecticide treated nets at antenatal clinic visits, and even management of other conditions such as COVID-19. Another adaptation includes systematically collecting and utilizing information gathered during OTSS visits through a routine Malaria Services and Data Quality Improvement initiative that incorporates data quality and client satisfaction.

Reporting on an independent evaluation of OTSS in four countries, Ashton et al.^7^ confirmed substantial improvement in health worker competencies to deliver quality malaria services including diagnosis, clinical management, and prevention. Health workers who received more OTSS visits attained the highest level of competence. The independent evaluation also included an 11-country qualitative evaluation of OTSS^8^ that established a high level of satisfaction among health providers, supervisors, and other stakeholders. Key features that were especially valued included the adaptability of the strategy package, the amount and quality of interaction between providers and supervisors with a focus on joint problem solving and action planning, and the digitization of checklists to guide the OTSS visits and streamline data collection and use. Participants broadly perceived OTSS to be effective at improving knowledge, skills, and service delivery. These independent findings confirmed evidence collected directly by the Impact Malaria teams and their malaria program colleagues. In Niger, Koko et al.^9^ used data collected on OTSS supervisor checklists to demonstrate improvements in both facility readiness predictors and quality service delivery outcomes for managing patients with fever or conducting antenatal consultations. Working across three countries, Bernard et al.^10^ demonstrated how facility readiness predicts health worker competence and can improve the quality of care. Other contributions to the supplement examined the impact of the OTSS package for improving malaria services for pregnant women,^11^^,^^12^ enhancing the community management of febrile illness,^13^ and advocating effectively to improve the management of severe malaria in hospital settings.^14^

Together, these accounts demonstrate how an approach based on multiple integrated implementation strategies can improve facility readiness and health worker competence to deliver quality malaria services. The evolution of the approach, its expansion to address facility-based case management as well as other malaria interventions, and its success across multiple transmission settings and health systems in nearly a dozen countries attest to the adaptability of OTSS based on local needs and priorities. By emphasizing OTSS consistently across multiple funding cycles, PMI, Impact Malaria, and their national malaria program partners have also demonstrated how quality continues to improve over time as health facilities and providers receive additional visits. These are lessons that will inform the scale-up and sustainability of the approach. Although the reports in this supplement stop short of documenting health impact at a population level, they answer critical questions about how to deliver malaria interventions that are well proven and recommended.

Since 2001, scale up of malaria interventions has had a substantial impact, reducing prevalence of infection by 40% across the African continent by 2015.^15^ Although the major part of this impact was attributed to massive campaigns distributing bed nets, improved case management still accounted for 19% of this historic progress, and its relative contribution was highly dependent on the coverage of prompt, effective treatment.^15^ A major challenge to improving the coverage and quality of malaria services is that information hasn’t regularly been collected to guide or monitor relevant outcomes, apart from national surveys at the household and health facility levels. Even when questions about care seeking or quality-of-care indicators are included in demographic and health surveys, malaria indicator surveys, and service provision assessments, these are limited, occurring at irregular intervals of 3 to 5 years or longer and only in specific geographic areas. In addition, population-based surveys nearly always use childhood fever as a proxy for malaria, an approach that was devised before universal parasitological diagnosis became a standard of care. They have been revised since, only to include limited details about the care those children received, such as whether they received a finger prick or heel stick, and whether they received an ACT. These measures are further limited by patients’ or parents’ recall and reporting. More contemporary standard measures reflecting current malaria case management practice have not been developed to monitor or improve malaria diagnosis and treatment.

The availability of routine parasitological diagnosis is beginning to transform health information systems by providing data on testing rates and confirmed malaria cases. These data are enabling endemic country health officials to track malaria burden at subnational levels and tailor their interventions based on local trends. Global malaria partners have responded by issuing guidance for subnational targeted programs. But these routine systems still fail to capture information on the quality of case management. They also seldom capture information about the age (apart from disaggregating cases among children under 5 years), gender, pregnancy status, or travel history of respondents. Malaria programs interested in cases among school-age children, pregnant women, or young men who may have acquired malaria while migrating for employment have had to devise parallel systems to track trends in these populations. In many malaria surveillance evaluations, only cases that receive a parasitological test are reported as suspected cases, making it difficult for program managers to monitor whether all potential cases have been tested.

The contributions from PMI Impact Malaria partners in this supplement demonstrate one approach to overcoming the limitations of routine surveillance and intermittent national surveys. The checklists that supervisors completed during OTSS visits have been exploited as sources of more continuous data on the fidelity of case management service delivery, collected from all participating health facilities. In doing so, the PMI Impact Malaria experience with OTSS may be much more than a promising quality improvement initiative adapted and applied in settings across multiple countries. It also suggests a model for monitoring the quality and availability of malaria services, something malaria programs across endemic, eliminating, and malaria-free countries can incorporate into their own supervision and support strategies. Individual and composite indicators of readiness and competence derived from supervision checklists allow for comparisons of quality services across settings and over time. Such indicators have been lacking as the nature of malaria case management has evolved over the past few decades. Scaling up OTSS and approaches like it stand to address persisting gaps in service quality as well as malaria information systems that incorporate indicators addressing current case management standard practices.
